# The role of Zn^2+^ in shaping intracellular Ca^2+^ dynamics in the heart

**DOI:** 10.1085/jgp.202213206

**Published:** 2023-06-16

**Authors:** Amy M. Dorward, Alan J. Stewart, Samantha J. Pitt

**Affiliations:** 1 School of Medicine, University of St Andrews, St Andrews, UK

## Abstract

Increasing evidence suggests that Zn^2+^ acts as a second messenger capable of transducing extracellular stimuli into intracellular signaling events. The importance of Zn^2+^ as a signaling molecule in cardiovascular functioning is gaining traction. In the heart, Zn^2+^ plays important roles in excitation–contraction (EC) coupling, excitation–transcription coupling, and cardiac ventricular morphogenesis. Zn^2+^ homeostasis in cardiac tissue is tightly regulated through the action of a combination of transporters, buffers, and sensors. Zn^2+^ mishandling is a common feature of various cardiovascular diseases. However, the precise mechanisms controlling the intracellular distribution of Zn^2+^ and its variations during normal cardiac function and during pathological conditions are not fully understood. In this review, we consider the major pathways by which the concentration of intracellular Zn^2+^ is regulated in the heart, the role of Zn^2+^ in EC coupling, and discuss how Zn^2+^ dyshomeostasis resulting from altered expression levels and efficacy of Zn^2+^ regulatory proteins are key drivers in the progression of cardiac dysfunction.

## Teaser

In this review, we discuss the role of Zn^2+^ and zinc transporters in regulating cellular Ca^2+^ dynamics in cardiac muscle.

## Introduction

Zinc is an essential trace element that is proposed to interact with >10% of the human proteome (Andreini et al., 2006). It is essential for processes including cell division (MacDonald, 2000) and protein synthesis (Kimball et al., 1995). The human body contains approximately 2–3 g of zinc. Of this, ∼60% is contained in skeletal muscle, ∼30% in bone, ∼5% in liver and skin, with the remainder distributed in other tissues, with ∼0.4% total zinc in the heart (reviewed in Jackson, 1989; Kambe et al., 2015). More than 99% of intracellular zinc is bound to proteins, although increasing evidence suggests that exchangeable zinc ions (Zn^2+^) act as second messengers capable of transducing extracellular stimuli into intracellular signaling events (Yamasaki et al., 2007). As more tools become available to study Zn^2+^, the importance and complexity of intracellular Zn^2+^ signaling are beginning to rival that of calcium ions (Ca^2+^), with key roles for Zn^2+^ evident in regulating many cellular processes. This review will focus on research specific to the cardiovascular system with a focus on the role of intracellular Zn^2+^.

Zn^2+^ plays an emerging but important role in heart function, including excitation–contraction (EC) coupling (Turan et al., 1997; Tuncay et al., 2011; Woodier et al., 2015; Reilly-O’Donnell et al., 2017), excitation–transcription coupling (Atar et al., 1995), and cardiac ventricular morphogenesis (Lin et al., 2018). In the heart, [Zn^2+^]_i_ is tightly regulated to maintain low labile Zn^2+^ concentrations. Hara et al. (2017) report the total extracellular [Zn^2+^] to range from 10 µM to high micromolar concentations, while the total intracellular [Zn^2+^] in mammalian cells is around 200 µM. Intracellular free Zn^2+^ concentrations are much lower than values reported for total Zn^2+^ and are cell-type dependent (reviewed by Vallee and Falchuk, 1993; Hara et al., 2017). If the exchangeable Zn^2+^ concentration moves outside a narrow range, either in excess or deficiency, this results in cardiac dysfunction, including altered contractile force (for reviews on this topic, see Pitt and Stewart, 2015; Stewart and Pitt, 2015; Turan and Tuncay, 2017). This highlights the importance of controlled Zn^2+^ homeostasis in cardiovascular functioning.

At rest, cardiomyocytes contain a small but measurable pool of free Zn^2+^ in the cytosol, reported to be between 100 pM and 2 nM. Certain triggers can lead to the release of Zn^2+^ from proteins and intracellular pools, and this can result in myocardial damage (Turan et al., 1997; Chabosseau et al., 2014). Little is known about the precise mechanisms controlling the intracellular distribution of Zn^2+^ and its variations during cardiac functioning. In this review, we consider the major pathways by which [Zn^2+^]_i_ is regulated in the heart, the role of Zn^2+^ in EC coupling, and how Zn^2+^ dyshomeostasis results in cardiac dysfunction.

## Zn^2+^ homeostasis in cardiomyocytes

### Zinc-binding proteins

Extracellular zinc speciation is a critical factor for Zn^2+^ uptake by all cells, irrespective of the tight control maintained through the action of transporter proteins. This is exemplified by recent work where ^68^Zn was used to measure zinc flux in immortalized endothelial cells (Coverdale et al., 2022). The concentration of serum albumin in the media was found to impact the rate of Zn^2+^ influx. This dynamic is of particular importance as serum albumin is the major carrier of plasma Zn^2+^ in circulation (Lu et al., 2008). In the absence of albumin under the conditions examined (20 μM ^68^Zn^2+^), the cells were unable to control the amount of Zn^2+^ taken up. This was indicated by an increase in total zinc within the cells over time, which was not observed when albumin was present in the media (Coverdale et al., 2022). Note that these findings are consistent with an earlier study that found the serum content of the extracellular media to be important for protecting cells of various types from otherwise harmful concentrations of Zn^2+^ (Haase et al., 2015). With relevance to the heart, it is suggested that low serum albumin levels in both males and females are associated with increased risk of myocardial infarction and linked to adverse outcomes after myocardial infarction. However, this topic remains controversial (Djoussé et al., 2002; Toida et al., 2020; Yoshioka et al., 2020).

Intracellular Zn^2+^ buffering in cardiomyocytes is tightly controlled by metallothioneins (MTs). MTs are low-molecular-weight, cysteine-rich proteins that play important roles in metal homeostasis and in the protection against intracellular heavy metal toxicity and oxidative stress at levels sufficient to induce cell damage. In humans, there are four main MT isoforms (MT1, MT2, MT3, and MT4) that are encoded by genes located on chromosome 16q13 (Thirumoorthy et al., 2011). Each MT protein can bind up to seven Zn^2+^ ions with high affinity, and collectively, MTs are thought to gather about 5–15% of the cytosolic zinc pool (Coyle et al., 2002). MTs work as zinc acceptors and donors to exchange Zn^2+^ with other proteins in the cells via oxidoreduction (Krężel and Maret, 2007). The thiol groups that coordinate zinc in MTs are redox reactive such that oxidation leads to the release of Zn^2+^. Basal levels of MTs in cells are often low, although they vary across different tissue types and their expression levels can be altered under certain conditions or disease states (Davis and Cousins, 2000). MT2A is the most abundant isoform found in heart, smooth muscle, and endothelial cells, whereas MT1E and MT1X are also significantly expressed in these tissues, suggesting these isoforms collectively play important roles in cardiovascular physiology (Choi et al., 2018).

### Zinc transporters expressed in the sarco/endoplasmic reticulum (S/ER)

**Figure 1.**
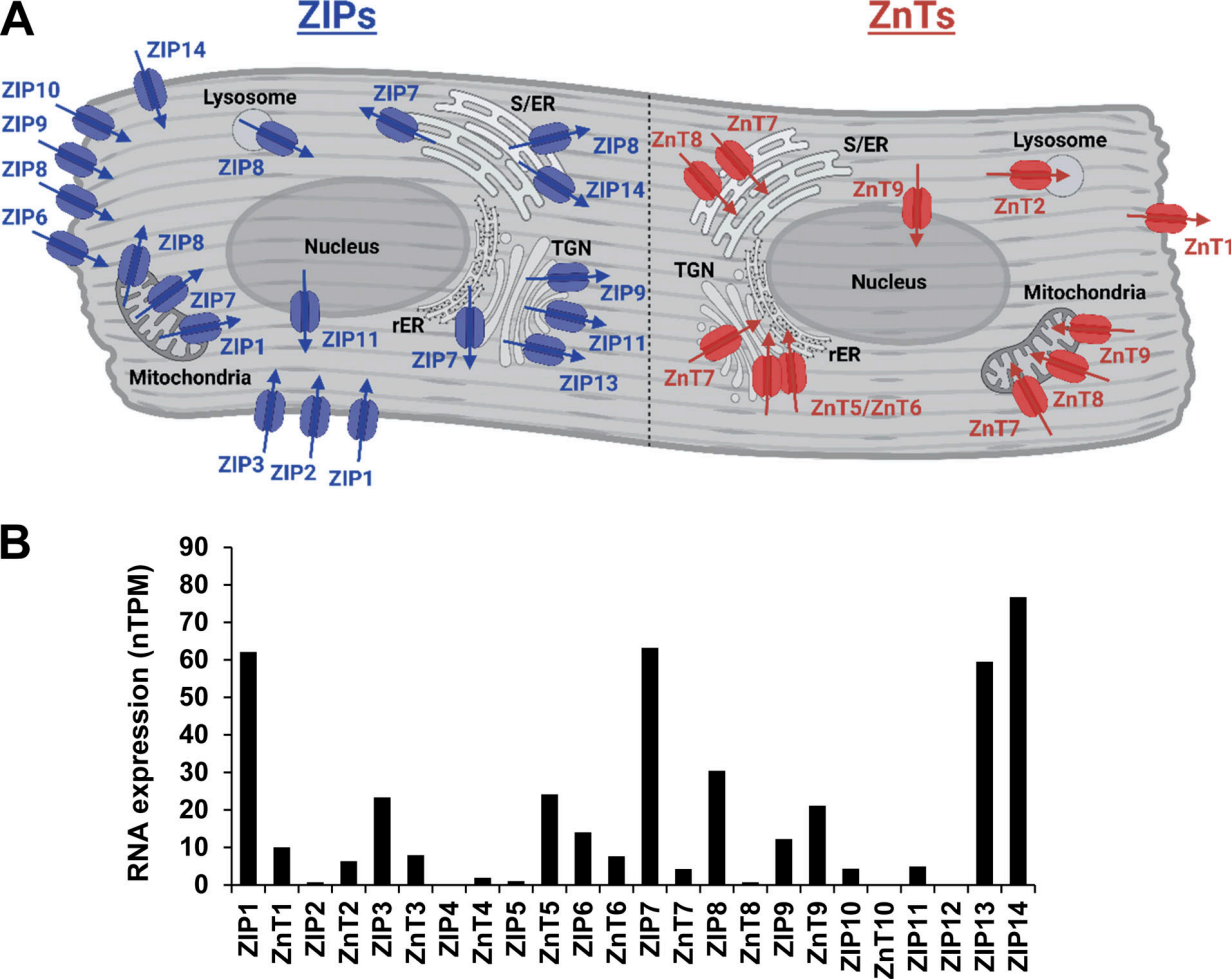
**Zn**^**2+**^ **transporters in the heart. (A)** Localization of zinc transporters in the heart. ZIP transporters are illustrated in blue on the left of the image while ZnT transporters are colored in red on the right of the image. Transporters with confirmed protein expression through the Human Protein Atlas or reported in published Western blot/immunofluorescent in heart tissue homogenates, isolated cardiomyocytes, or cardiac cell lines (such as H9C2 cells) were included. rER, rough ER; TGN, trans-Golgi network. Created with BioRender.com. **(B)** RNA expression of Zn^2+^ transporters in normalized protein-coding transcripts per million (nTPM) in human heart. Figure was created using information available from the Human Protein Atlas (2022), Uhlén et al. (2015), and Choi et al. (2018).

The movement of Zn^2+^ across cell membranes is facilitated by zinc transporters. There are 24 known zinc transporters in humans, which are classified into two groups: zinc transporters (ZnTs; 1–10) designated to the solute carrier family 30A (SLC30A) and zrt-, irt-related proteins (ZIPs; 1–14), grouped as solute carrier family 39A (SLC39A; Paulsen and Saier, 1997; Grotz et al., 1998; Eide, 2004; Palmiter and Huang, 2004; Cousins et al., 2006). ZnTs transport Zn^2+^ from the cytosol into organelles or to the extracellular space, while ZIPs transport Zn^2+^ into the cell from the extracellular matrix or from organelles into the cytosol (Conklin et al., 1994; Palmiter and Findley, 1995; Taylor, 2000; Taylor et al., 2003). Zn^2+^ can also be transported through Ca^2+^ channels, including the L-type calcium channel (LTCC) in cardiomyocytes (Atar et al., 1995). The expression profile of zinc transporters within the heart are shown in Table 1 (ZIPs) and Table 2 (ZnTs). The localization of these zinc transporters is illustrated in Fig. 1 A, while Table 3 details the localization and detection method. Fig. 1 B shows RNA expression of ZIPs and ZnTs in heart. An increase in intracellular Zn^2+^ leads to metal regulatory transcription factor 1 (MTF-1) binding, resulting in MTF-1 translocation to the nucleus and subsequent activation to bind DNA and initiate MT expression (Bittel et al., 1998). It is suggested that Zn^2+^ sequestration into organelles is the first response to Zn^2+^ influx to deal with the potential threat of a harmful increase in cytosolic Zn^2+^ while transcription and translation of zinc transporters and MTs occur (Kukic et al., 2014).

**Table 1.** Protein expression (score) of ZIPs in heart tissue

	ZIP1	ZIP2	ZIP3	ZIP4	ZIP5	ZIP6	ZIP7	ZIP8	ZIP9	ZIP10	ZIP11	ZIP12	ZIP13	ZIP14
Heart	N/A	Low	High	N/A	N/A	Med	Med	Low	Med	Low	N/A	N/A	ND	Med

Score ranged from high to not detected (ND). N/A illustrates transporters on the atlas which are pending normal tissue analysis. Data obtained from Uhlén et al. (2015) and Human Protein Atlas (2022).

**Table 2.** Protein expression (score) of ZnTs in heart tissue

	ZnT1	ZnT2	ZnT3	ZnT4	ZnT5	ZnT6	ZnT7	ZnT8	ZnT9	ZnT10
Heart	Low	N/A	ND	N/A	Med	Low	Med	ND	Med	ND

Score ranged from high to not detected (ND). N/A illustrates transporters on the atlas which are pending normal tissue analysis. Data obtained from Uhlén et al. (2015) and Human Protein Atlas (2022).

**Table 3.** Subcellular localization of zinc transporters

Zinc transporter	Localization	Detection Method			Reference
		Immunofluorescence	Cell fractionation and immunoblotting	Zn^2+^ influx/efflux assay/measurement of [Zn^2+^]	
ZIP1	PM	✓		✓	Gaither and Eide, 2001
	Mitochondria	✓	✓		Cho et al., 2019
ZIP2	PM	✓		✓	Gaither and Eide, 2000
ZIP3	PM	✓			Kelleher and Lönnerdal, 2003
ZIP6	PM	✓			Taylor and Nicholson, 2003
ZIP7	TGN	✓		✓	Huang et al., 2005
	S/ER	✓	✓	✓	Tuncay et al., 2017
	Mitochondria	✓	✓	✓	Tuncay et al., 2019
ZIP8	PM	✓	✓		Dalton et al., 2005
	Lysosomes	✓			Aydemir et al., 2009
	Mitochondria S/ER		✓ ✓		Olgar et al., 2019
ZIP9	PM	✓	✓	✓	Thomas et al., 2014
	TGN	✓			Matsuura et al., 2009
ZIP10	PM	✓	✓		Lichten et al., 2011
ZIP11	TGN	✓			Kelleher et al., 2012
	Nucleus	✓	✓		Martin et al., 2013
ZIP13	TGN	✓		✓	Fukada et al., 2008
ZIP14	PM	✓			Taylor et al., 2003
	S/ER	✓			Olgar et al., 2018a
ZnT1	PM			✓	Palmiter and Findley, 1995
ZnT2	Lysosomes	✓			Palmiter et al., 1996
ZnT5	TGN	✓	✓	✓	Kambe et al., 2002
ZnT6	TGN	✓	✓		Suzuki et al., 2005
ZnT7	TGN	✓			Kirschke and Huang, 2003
	S/ER	✓	✓		Tuncay et al., 2017
	Mitochondria	✓	✓		Tuncay et al., 2019
ZnT8	Mitochondria S/ER		✓ ✓		Olgar et al., 2019
ZnT9	Nucleus	✓	✓		Sim and Chow, 1999
	Mitochondria	✓			Kowalczyk et al., 2021

Subcellular localization of ZIPs and ZnTs as illustrated in Fig. 1 A. PM, plasma membrane; TGN, trans-Golgi network.

**Figure 2.**
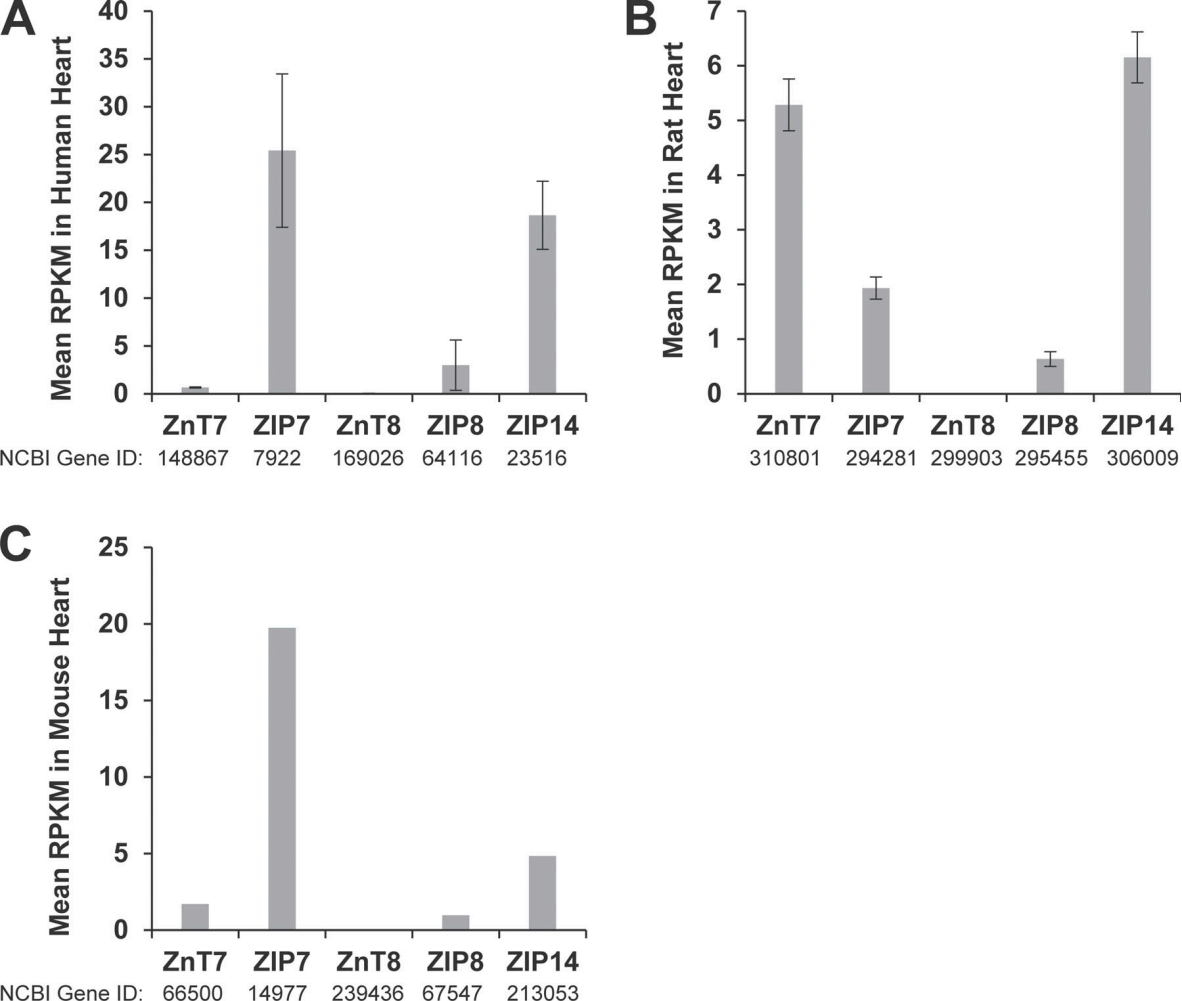
**RNA expression of S/ER-located Zn**^**2+**^ **transporters. (A)** Mean reads per kilobase of transcript per million reads mapped (RPKM) of Zn^2+^ transporters in human heart (RNA sequencing [RNA-Seq] data from Fagerberg et al., 2014). **(B)** Mean RPKM of Zn^2+^ transporters in rat heart (21 wk; RNA-Seq data from Yu et al., 2014). **(C)** Mean RPKM of Zn^2+^ transporters in mouse heart (RNA-Seq data from Yue et al., 2014).

Numerous organelles have been identified as Zn^2+^ stores, as described below. While the S/ER is classically known as a Ca^2+^ store, Zn^2+^ is also stored in this organelle. Using genetically encoded Zn^2+^ sensors, the labile Zn^2+^ concentration in the S/ER has been estimated to be between 1 pM and ≥5 nM (Qin et al., 2011; Chabosseau et al., 2014). There are numerous proteins in the S/ER that bind Zn^2+^, including calsequestrin 2 (CSQ2) and calreticulin, which also bind Ca^2+^ (Baksh et al., 1995; Tan et al., 2006). The S/ER has Zn^2+^ transporters within its membrane. Localization of ZnT7 and ZIP7 to the S/ER was first demonstrated in the heart by Tuncay et al. (2017). Turan and co-workers also subsequently reported localization of ZIP8, ZIP14, and ZnT8 to the S/ER in H9C2 cells (embryonic rat myoblasts; Olgar et al., 2018a), but ZnT8 has not yet been detected at the gene level (Fig. 2).

Zn^2+^ can be sequestered within other cell organelles. Labile Zn^2+^ is undetectable in the nucleus, even though it is estimated that 30–40% of total cellular Zn^2+^ resides in the nucleus (Vallee and Falchuk, 1993; Lu et al., 2016). The Golgi is estimated to contain between 0.2 pM and 25.1 nM free Zn^2+^, while the mitochondria are estimated to contain between 0.14 and 300 pM Zn^2+^ (Qin et al., 2011; Park et al., 2012; McCranor et al., 2012; Chabosseau et al., 2014; Kowada et al., 2020). Lysosomes have also been identified as Zn^2+^ stores, although the concentration in these organelles has not yet been determined (Roh et al., 2012; Kukic et al., 2014).

### Organelle crosstalk shapes Ca^2+^ and Zn^2+^ signaling

The importance of communication between cellular organelles and exchange of messenger molecules is well established (reviewed by Rossini et al., 2021). Membrane-contact sites regulate many cellular functions. In the heart, dysregulation of different organellar crosstalk pathways results in pathology (reviewed by Dabravolski et al., 2022; Hulsurkar et al., 2022). Some examples of organellar crosstalk between Ca^2+^ and Zn^2+^ are provided below.

Mitochondria and S/ER actively communicate with each other to promote a variety of cellular events. Mitochondria play multiple roles in cardiac cells, including regulation of energy homeostasis, signaling, metabolism, and cell death pathways. Crosstalk between the SR and mitochondria is important in normal cardiomyocyte viability and EC coupling and plays a key role in regulating Ca^2+^-signaling responses in cardiac muscle (Griffiths and Rutter, 2009; Eisner et al., 2013). While the SR and mitochondria are separate compartments with different functions, the interplay between the SR and mitochondria is essential in supporting cardiomyocyte contraction and relaxation, and this organellar crosstalk facilitates adaptation to changing metabolic demands during EC coupling (Dorn II and Maack, 2013; Gorski et al., 2015).

Mitochondria have also been identified as intracellular Zn^2+^ stores. Mitochondrial-free [Zn^2+^] is maintained at lower concentrations than found in the cytosol (Ye et al., 2001; Kambe et al., 2015). Emerging research suggests that in cardiomyocytes, the interplay between Zn^2+^ homeostasis and crosstalk between the mitochondria and S/ER is important in cardiovascular diseases (for a recent review, see Dabravolski et al., 2022). Close contact between the ER and mitochondria was first described by Vance, who through fractionation, identified a pool of phospholipids that were suggested to be involved in the association of the ER and mitochondria (Vance, 1990). These mitochondria-associated membranes (MAMs) are the site at which the mitochondria and ER communicate functionally and through structural interaction (reviewed in Giorgi et al., 2009). The role of MAMs in cardiovascular disease is reviewed in detail by Wang et al. (2021b). It is thought that intracellular Ca^2+^ machinery including the inositol 1,4,5-trisphosphate receptor (IP3R) may be involved in Ca^2+^ signaling across the mitochondria and ER (Hirota et al., 1999). Emerging evidence suggests that this may also be the case with Zn^2+^.

Work from the Turan group illustrates that in aged rats, aged-related increase in intracellular [Zn^2+^] is reduced using antioxidant MitoTEMPO, while age-related alterations in mitochondrial ZIP7, ZIP8, and ZnT8 are reversed by MitoTEMPO treatment (Olgar et al., 2019). They also illustrate that key proteins involved in S/ER-mitochondrial coupling including mitofusin-protein (Mfn-1/2), mitochondrial fission protein (Fis-1), and S/ER-mitochondrial bridge protein B cell receptor associated protein 31 are significantly altered when ZIP7 was silenced in high glucose and doxorubicin-treated H9C2 cells (Tuncay et al., 2019). Protein expression of stromal interaction molecule 1 (STIM1), a S/ER Ca^2+^ sensor that regulates store-operated calcium entry, is also significantly altered in hyperglycaemic and doxorubicin-treated H9C2 cells (Tuncay et al., 2019). In cardiomyocytes, it is suggested that STIM1 contributes to the development of cardiac hypertrophy and advancement of heart disease, although how STIM1 expression and functionality impact S/ER Zn^2+^ and Zn^2+^ transporters has not yet been investigated (Bootman and Rietdorf, 2017). Tight coupling between Ca^2+^ and Zn^2+^ dynamics is also important for regulation of cellular functions in the heart. Research by Kamalov and colleagues showed that these ions are intrinsically coupled in aldosterone-treated rat hearts, suggesting their crosstalk contributes to altering the redox state of the cardiomyocytes (Kamalov et al., 2009).

In the nucleus, Zn^2+^ plays an important role in gene transcription and in maintaining the stability of DNA through zinc-finger proteins, with Zn^2+^ deficiency leading to a reduction in DNA repair and compromise of integrity due to destabilization of DNA (Ho, 2004). The effect of nuclear Zn^2+^ dyshomeostasis on the heart/cardiovascular system has to our knowledge not yet been investigated. Zn^2+^ and zinc transporters have also been linked to lysosome function and cellular autophagy in breast tissue and neuronal cell types (Rivera et al., 2018; Kim et al., 2022). In human embryonic kidney (HEK293) cells, Cuajungco and colleagues suggest association of zinc transporter transmembrane protein 163 (TMEM163) and cation channel transient receptor potential mucolipin 1 (TRPML1) is essential for Zn^2+^ homeostasis and disruption to this association may be a mechanism for Zn^2+^ overload in mucolipidosis type IV disease, a genetic neurodevelopmental disorder (Cuajungco et al., 2014). It is suggested that TRPLM1 agonists lead to cell death through a Zn^2+^-dependent lysosomal pathway with mitochondrial swelling in metastatic melanoma cells (Du et al., 2021). Interaction of Zn^2+^/zinc transporters and TRPLM1 has not been investigated in the heart; however, Li and Li have reviewed the role of TRPLM1 and Ca^2+^ in cardiovascular diseases (Li and Li, 2021).

### Coupling of Zn^2+^ and Ca^2+^ homeostasis in the heart

Different divalent cations can often bind to the same or similar binding sites in proteins. In general, Ca^2+^ and Mg^2+^ favor protein binding sites composed of O-ligands (for example, aspartic acid or glutamic acid sidechains), whereas Zn^2+^ favors protein binding sites that additionally possess N- and S-ligands (for example, histidine and cysteine sidechains, respectively; reviewed by Vallee and Auld, 1990; Alberts et al., 1998; Bindreither and Lackner, 2009; Tang and Yang, 2013). Zn^2+^ sites are typically of a lower coordination number than Ca^2+^ or Mg^2+^ sites (Bock et al., 1995). While a limited degree of overlap does exist (Zn^2+^ also can bind aspartic acid and glutamic acid residues), it is important to point out that Zn^2+^ is typically present (both intracellularly and extracellularly) at a lower concentration than Ca^2+^ and Mg^2+^. This, together with the respective affinity of a particular site/region for each metal determines which will bind (or whether competition between different metals may occur). We have previously shown that the type-2 ryanodine receptor (RyR2) has both high-affinity Zn^2+^ activation sites and low-affinity Zn^2+^ inhibition sites. Although the inhibitory action of Zn^2+^ is likely a consequence of Zn^2+^ binding to the divalent inhibitory site of the channel, at least some of the activatory sites are distinct from the Ca^2+^ binding sites (Woodier et al., 2015).

As well as ion channels, intracellular proteins are also capable of binding both Ca^2+^ and Zn^2+^. One example of this is CSQ2, a Ca^2+^-binding protein located in the S/ER, important in Ca^2+^ regulation of RyR2 (Meissner and Henderson, 1987). CSQ2 has been shown to bind both Ca^2+^ and Zn^2+^, while Zn^2+^ is thought to modulate the function and structure of CSQ2 (Baksh et al., 1995). Baksh and colleagues report that CSQ2 has a large Ca^2+^-binding capacity (∼40–50 mol of Ca^2+^ per mole protein) with moderate affinity (average *K*_d_ ≈ 1 mM; Baksh et al., 1995). For Zn^2+^, the binding capacity is much higher (∼200 mol of Zn^2+^ per mole protein) exhibiting an average *K*_d_ ≈ 300 µM (Baksh et al., 1995). It is not known if CSQ2 binds Ca^2+^ and Zn^2+^ at the same sites; however, other Ca^2+^ proteins which also bind Zn^2+^, such as histidine-rich Ca^2+^-binding protein in skeletal muscle and calmodulin in the brain, possess separate Zn^2+^ and Ca^2+^ binding sites (Baudier et al., 1983; Picello et al., 1992). Furthermore, Zn^2+^-binding at Ca^2+^-effector sites in certain proteins may be unable to induce the same structural changes. For example, in a study by Warren and co-workers, it was shown that when Zn^2+^ bound to the EF-hand motif of calmodulin, the overall structure of the zinc-bound form resembled the apo-form rather than the calcium-bound form (Warren et al., 2007).

The interaction of Ca^2+^ and Zn^2+^ is not a novel concept. Yamasaki and colleagues report that Zn^2+^ release in mast cells from the S/ER, in the form of a Zn^2+^ wave, was Ca^2+^ dependent (Yamasaki et al., 2007). G protein-coupled receptor 39 (GPR39) was identified to be stimulated by Zn^2+^ by Holst et al. (2007) and the receptor is now often referred to as the Zn^2+^-sensing receptor. GPR39 is located on the plasma membrane and is thought to act as an extracellular Zn^2+^ sensor to trigger activation of several G protein-coupled pathways, including the mobilization of intracellular Ca^2+^ through G_q_ coupling (Popovics and Stewart, 2011). The presence of a cellular zinc receptor with the ability to trigger Ca^2+^ release had much earlier been reported by Hershfinkel et al. (2001). With relevance to G protein-coupled receptors (GPCRs), work by Hojyo and colleagues utilized *Slc39a14*-knockout mice to implicate ZIP14 in GPCR signaling, where it was found that mice that lack the ZIP14 transporter display restricted growth (Hojyo et al., 2011). In the heart, GPCR signaling can influence intracellular Ca^2+^ signaling, leading to altered cardiac contractility and cardiomyocyte apoptosis (Communal et al., 1999; Nash et al., 2001). While the influence of GPCRs will not be discussed further in this review, Salazar et al. (2007) and Wang et al. (2018) have reviewed cardiac GPCRs and the role of GPCRs in cardiovascular disease.

In 1995, Atar and colleagues demonstrated through use of live cell imaging and electrophysiology that Zn^2+^ could enter rat cardiac muscle through the LTCC (Atar et al., 1995). While the role of the LTCC in Ca^2+^ handling is well established in EC coupling, little is known about the interaction between LTCCs and Zn^2+^ in the heart (Bodi et al., 2005). However, in the brain, it was demonstrated that Zn^2+^ accumulation can occur in astrocytes (a subtype of glial cells in the brain) through LTCC in a manner that is attenuated by ZnT1 (Nolte et al., 2004). A subsequent publication by the same group reported that ZnT1 can regulate Zn^2+^ and Ca^2+^ permeation through LTCC in HEK293 cells. In these cells, expression of ZnT1 reduced Ca^2+^ influx by ∼40% (Segal et al., 2004). The Moran laboratory has shown that ZnT1 is also capable of inhibiting LTCC (Beharier et al., 2007; Beharier et al., 2010; Levy et al., 2009). This work shows that crosstalk between ion channels and transporters can influence the cellular movement of ions, which suggests that the interaction of LTCC and ZnT1 can influence cardiac function. Increased ZnT1 protein expression as a result of rapid pacing in culture cardiomyocytes is suggested to lead to reduced Ca^2+^ influx through LTCC and contribute to atrial fibrillation in atrial tachycardia (Beharier et al., 2010). Recent research by Wang et al. (2021a) has highlighted a link between Ca^2+^ signaling and the expression of Zn^2+^ transporters. Using a cellular model of ischemia/reperfusion (I/R) involving H9C2 cells and isolated murine cardiomyocytes in combination with Ca^2+^ and Zn^2+^ chelators, the group reported that Ca^2+^ mobilization triggers a reduction in ZIP13 protein expression. This reduction of ZIP13 was reported to activate Ca^2+^/calmodulin-dependent protein kinase II and contribute to I/R injury.

Transient receptor potential kinase ankyrin 1 (TRPA1) is located on the S/ER in cardiac cells, has also been linked to intracellular Ca^2+^ movement, and is implicated in atherosclerosis and heart failure (reviewed by Wang et al., 2019). In neurons, TRPA1 has been shown to be Zn^2+^-activated at [Zn^2+^] of 300 nM and inhibitory at [Zn^2+^] >300 µM (Hu et al., 2009). As well as being Ca^2+^ permeable, TRPA1 is also Zn^2+^ permeable. The interaction between Zn^2+^ and Ca^2+^ and its impact on vascular tone regulation has been recently reported by Betrie et al. (2021). However, this has not been investigated in the heart. TRPML1, transient receptor potential mucolipin 7, and transient receptor potential cation channel subfamily C member 6 are also present in the heart, have been linked to cardiac pathologies, and are permeable to both Ca^2+^ and Zn^2+^ (reviewed by Bouron et al., 2015).

### Actions of Zn^2+^ during EC coupling

Cardiac EC coupling is a process that governs contractility of the heart through carefully controlled release of Ca^2+^ from the S/ER. An action potential travels down the transverse tubule of a cardiomyocyte where depolarization activates LTCCs, leading to Ca^2+^ influx (Bers, 2002). The resulting [Ca^2+^] in the dyadic cleft—the intracellular space between the plasma membrane and SR—increases to >10 μM, leading to activation of localized RyR2s on the SR membrane (Bers, 2002). This increase in cytosolic [Ca^2+^] causes activation of multiple proximal RyR2 channels in a process termed calcium-induced calcium release (Fabiato, 1983). Recruitment of RyR2 molecules and their synchronous activation is necessary for a Ca^2+^ release event from the SR to occur (Zima et al., 2010). At low micromolar levels, intracellular Ca^2+^ binds to troponin C of the troponin complex, causing troponin I inhibition and initiating a conformational change of the troponin–tropomyosin complex (de Tombe, 2003; Fearnley et al., 2011). This allows crossbridge formation between myosin and actin in the presence of ATP and leads to a power stroke in which ATP is hydrolyzed and the contractile machinery is activated. This translates into cardiac muscle contraction, termed systole (Bers, 2002; de Tombe, 2003). As such, disruption to Ca^2+^ handling during EC coupling results in impaired cardiac contractility and function.

The effects of Zn^2+^ on cardiomyocyte function are thought to involve a competitive effect of Zn^2+^ on Ca^2+^ regulatory mechanisms. In isolated cardiomyocytes, extracellular Zn^2+^ reduces cardiomyocyte contractile functioning (Ciofalo and Thomas, 1965; Yi et al., 2012; Yi et al., 2013) and this is thought to be a consequence of extracellular Zn^2+^ being able to act as a charge carrier through LTCC resulting in a 70% reduction in the inward Ca^2+^ current (Atar et al., 1995). Studies have shown that cardiomyocytes exposed to extracellular Zn^2+^ display a 50% reduction in S/ER calcium load (Turan 2003; Qin et al., 2011; Yi et al., 2012), revealing a relationship between intracellular organelles, intracellular Zn^2+^ dynamics, and intracellular Ca^2+^ movements.

### Zn^2+^-induced regulation of RyR2

RyR2 is the route through which Ca^2+^ is released from the S/ER providing the necessary driving force for cellular contraction. Interestingly, RyR2 discriminates only slightly between divalent cations (Tinker and Williams, 1992) and has been shown to be permeable to Mg^2+^, Sr^2+^, Ba^2+^ (Diaz-Sylvester et al., 2011), and very recently Zn^2+^ (Gaburjakova and Gaburjakova, 2022). This suggests that Zn^2+^ may contribute to the RyR2 current during EC coupling. Recent work has also suggested that even a very small Zn^2+^ current in the lumen-to-cytosol direction is sufficient to saturate the Zn^2+^ finger motif situated within the C-terminal tail of the four RyR2 subunits, and that binding of Zn^2+^ in this region is essential for RyR2 function (Gaburjakova and Gaburjakova, 2022). At the cellular level, Tuncay and co-workers showed ryanodine-sensitive Zn^2+^ transients with similar kinetics to Ca^2+^ in stimulated rat cardiomyocytes, providing further evidence that the S/ER is an intracellular Zn^2+^ pool and that Zn^2+^ levels are elevated during the cardiac cycle (Tuncay et al., 2011). They proposed that the rapid changes in free Zn^2+^ resulted from displacement by Ca^2+^ from intracellular binding sites that are highly sensitive to the redox status of the cardiomyocytes. It is not unreasonable to speculate that RyR2 also contributes to this Zn^2+^ signal.

Zn^2+^ release from the S/ER is unlikely to trigger contraction, but this small release of Zn^2+^ may be sufficient to shape Ca^2+^ dynamics in cardiomyocytes by amplifying the Ca^2+^ response through RyR2. In our own study, it was shown at the single-channel level that cytosolic Zn^2+^ can act as a high-affinity activator of RyR2 (Woodier et al., 2015). Concentrations of free Zn^2+^ ≤1 nM potentiated RyR2 activity but the presence of activating levels of cytosolic Ca^2+^ was a requirement for channel activation. However, at concentrations of Zn^2+^ >1 nM, the main activating ligand switched from Ca^2+^ to Zn^2+^, and the requirement of Ca^2+^ for channel activation was removed. The ability of Zn^2+^ at a concentration of 1 nM to directly activate RyR2 reveals that RyR2 has a much higher affinity for Zn^2+^ than Ca^2+^ (by approximately three orders of magnitude). We also showed that Zn^2+^ modulated both the frequency and amplitude of Ca^2+^ waves in cardiomyocytes in a concentration-dependent manner and that reduction of the [Ca^2+^]_i_ to subactivating concentrations failed to abolish Ca^2+^ waves in the presence of 1 nM Zn^2+^. These data suggest that RyR2-mediated Ca^2+^ homeostasis is intimately related to intracellular Zn^2+^ levels. In the heart, RyR2 channels operate in closely packed clusters (Baddeley et al., 2009; Hayashi et al., 2009; Sheard et al., 2022). It is conceivable that the Zn^2+^ current mediated through RyR2, although small, is sufficient to sensitize and recruit other RyR2 channels to help shape cellular Ca^2+^ responses. The role of Zn^2+^ as both a high-affinity activator of RyR2, modulator of channel function in the absence of Ca^2+^, and charge carrier that contributes to the RyR2-mediated current is a paradigm shift in our understanding of how RyR2 is activated during EC coupling. The recently identified role of ZnT1 as a neuronal Ca^2+^/Zn^2+^ transporter (Gottesman et al., 2022) opens the suggestion that Zn^2+^ is delivered to RyR2 by a zinc transporter located in the S/ER or the plasma membrane. However, further work is required to address this question. What is certain is that Zn^2+^ and Ca^2+^ dynamics are intrinsically coupled.

### Mitsugumin-23 as a putative Zn^2+^-regulated, Ca^2+^-permeable ion channel

**Figure 3.**
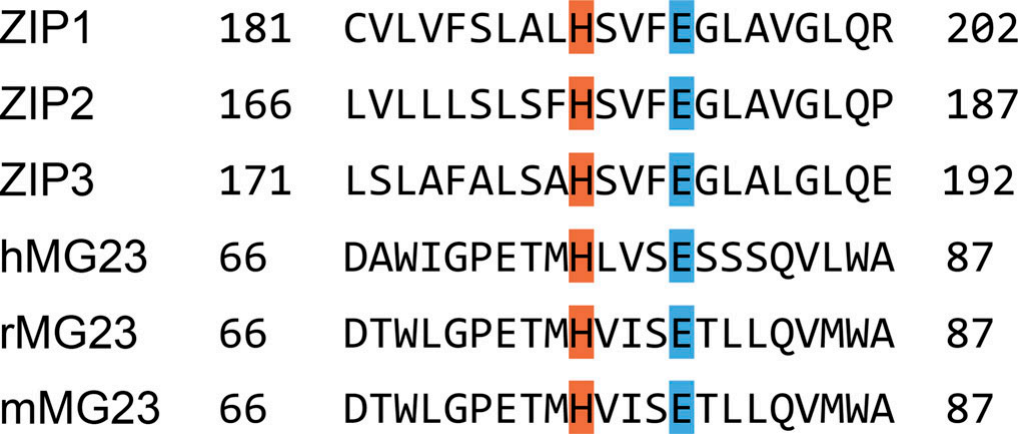
**Possible Zn**^**2+**^ **binding sites on MG23.** Partial sequence alignment of human zinc transporters ZIP1, ZIP2, and ZIP3 illustrating the conserved Zn^2+^ binding motif, H-x-x-x-E. Histidine residues (H) are highlighted in orange and glutamate (E) residues are highlighted in blue. This motif is also conserved across human (h), rat (r), and murine (m) MG23.

**Figure 4.**
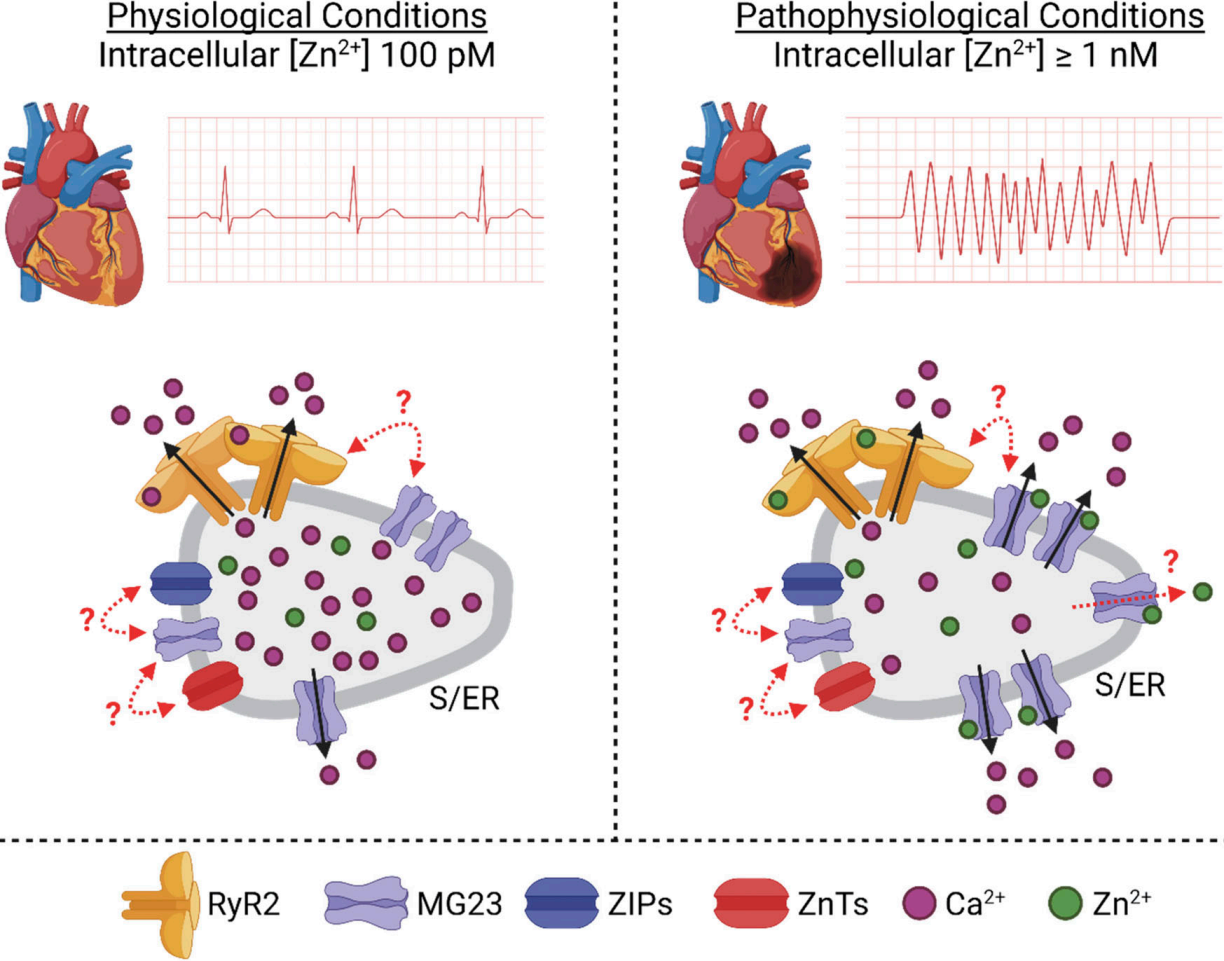
**Graphical summary of the suggested role of MG23 in cardiovascular function.** MG23 may contribute to the release of Ca^2+^ from S/ER Ca^2+^ stores. In pathophysiological conditions where intracellular Zn^2+^ is elevated, the activity of MG23 will be increased, leading to increased release of Ca^2+^ from the S/ER. Increased [Zn^2+^]_i_ will result in activation of RyR2. Dotted lines and question marks suggest putative interactions/functions. Figure created with BioRender.com.

RyR2 is not the only Ca^2+^-permeable ion channel localized to S/ER stores. TMEM109 or Mitsugumin-23 (MG23) is a 23-kD transmembrane protein found in the S/ER and nuclear membranes of cardiac muscle cells and other tissues including skeletal muscle, epithelial cells, and the brain (Nishi et al., 1998). MG23 is a voltage-sensitive non-selective cation channel. MG23 has an unusual morphology as shown by electron microscopy and 3-D particle reconstruction. Two types of particles were consistently observed: a small asymmetric particle composed of six homomeric subunits and a larger bowl-shaped particle forming a hexametric megastructure composed of six asymmetric particles (Venturi et al., 2011). The mega pore structure is hypothesized to readily assemble and disassemble, and this is functionally mirrored in the observed gating behavior of MG23. Recombinant purified MG23 proteins reconstituted into planar lipid bilayers exhibit very unusual gating behavior characterized by brief “flickery” opening events and coordinated gating of multiple channels (Venturi et al., 2011; Reilly-O’Donnell et al., 2017). It is likely that both the asymmetric particle and the megastructure permit ion permeation, and that the unusual gating behavior reflects the apparent instability of MG23. The MG23 channel has received little attention, but given its location and its ability to conduct Ca^2+^, it is likely that it contributes to the Ca^2+^ leak and/or Ca^2+^ current in cardiac cells. Information regarding modulators of MG23 activity is currently lacking but our recent work has shown that cytosolic Zn^2+^ increases MG23 activity (Reilly-O’Donnell et al., 2017). Glutamate, aspartate, histidine, and cysteine amino acid residues are commonly associated with Zn^2+^ binding sites. Surprisingly, human MG23 does not have any cysteine residues and so lacks the classic C2H2 zinc finger motif. MG23 does have a common conserved H-x-x-x-E sequence, which is attributed to Zn^2+^ binding in Zn^2+^ transporters including ZIP1, ZIP2, and ZIP3 (Fig. 3; Kambe et al., 2015). Hydrophobicity plots published by Nishi et al. (1998) suggest the part of the protein containing this sequence is localized in the SR lumen. It is not known whether RyR2 and MG23 interact with each other or if MG23 is part of the calcium release unit. One could speculate that the recently described RyR2-mediated Zn^2+^ current might trigger recruitment and initiation of MG23-mediated Ca^2+^ fluxes, as summarized in Fig. 4.

### Zn^2+^-induced regulation of IP_3_Rs

The role of IP_3_R in EC coupling is considered of most importance during early cardiac development (Luo et al., 2020). As the S/ER matures, the number of RyR2 channels increases and in adult cardiomyocytes RyR2 mRNA levels are ∼50-fold higher than IP_3_R (Moschella and Marks, 1993). Despite this, IP_3_Rs located in the nuclear envelope are involved in excitation–transcription coupling, thereby participating in the control of gene expression (Nakayama et al., 2010). In mammalian cardiomyocytes, Zn^2+^ plays a key role in excitation–transcription coupling where Zn^2+^ influx through LTCC mediates voltage-dependent gene expression (Atar et al., 1995), suggesting a possible link between Zn^2+^ and IP_3_R in regulation of gene expression. In dissociated rat hippocampal neuronal cultures, relatively small changes in cytosolic Zn^2+^ during stimulation altered expression levels of 931 genes with IP_3_R type-2 being markedly upregulated (Sanford et al., 2019). Zn^2+^ can be released from S/ER stores upon IP_3_R stimulation. The release of caged inositol 1,4,5-trisphosphate (IP_3_) in cultured cortical neurons resulted in the release of Zn^2+^ from thapsigargin-sensitive stores, suggesting that sequestration of Zn^2+^ into the S/ER is important in regulation of intracellular levels and that Zn^2+^ is released following agonist stimulation (Stork and Li, 2010). How Zn^2+^ modulates IP_3_ signaling in the heart is an underexplored area of research. Although to date there is no demonstration that IP_3_Rs are directly modulated by Zn^2+^, IP_3_Rs have a C2H2 zinc finger domain in the C-terminal tail that plays a critical role in regulation of channel activity (Furuichi et al., 1989). Individual or combined cysteine and histidine mutations within this conserved C2H2 domain resulted in the abolition of IP_3_R type-1 functioning (Uchida et al., 2003; Bhanumathy et al., 2012). This C2H2 C-terminal domain region is also highly conserved across the RyR family and is thought to be important in maintenance of RyR2-mediated Zn^2+^ currents (Gaburjakova and Gaburjakova, 2022), suggesting a fundamental role for Zn^2+^ in intracellular Ca^2+^ channel regulation and cellular Ca^2+^ dynamics.

## Dysregulation of cardiac Zn^2+^ homeostasis in disease

### Role of Zn^2+^-binding proteins in disease

The ability of serum albumin in the extracellular environment to bind and buffer Zn^2+^ is known to be compromised by the binding of fatty acids (Stewart et al., 2003; Lu et al., 2012; Sobczak et al., 2021a), which it transports through binding at up to seven different sites (Bhattacharya et al., 2000). Total plasma levels of fatty acids are generally quite low (<1 mol eq. relative to albumin; Sobczak et al., 2021a; Sobczak et al., 2021b) but can be elevated in some disease states. Although high plasma fatty acid levels are known to increase the risk of heart failure and sudden cardiac death (Pilz et al., 2007; Djoussé et al., 2013), how this dynamic might impact cellular Zn^2+^ uptake under physiological conditions has yet to be investigated.

Zn^2+^ supplementation is known to induce cardiac MT expression (Wang et al., 2006), emphasizing its importance in regulating zinc homeostasis in the heart. Several studies have highlighted a protective role for MTs in helping to prevent/reduce cardiomyopathy and oxidative stress. It has been shown that overexpression of MT in cell and animal models protects cardiomyocytes from diabetic cardiomyopathy (Liang et al., 2002; Cai et al., 2006; Huang et al., 2021). Cardiac-specific overexpression of MT reduces cigarette smoking exposure–induced myocardial contractility and mitochondrial damage (Hu et al., 2013). Zinc-induced MT expression has been shown to reduce doxorubicin-induced damage in cardiomyocytes (Kimura et al., 2000; Jing et al., 2016). In addition, alcohol-induced cardiac hypertrophy and fibrosis were observed in MT-knockout mice fed an alcohol-containing liquid diet for 2 mo but not in wild-type mice fed the same diet (Wang et al., 2005). Similarly, doxorubicin-induced cardiomyopathy was found to be more severe in MT-knockout mice in than wild-type mice (Kimura et al., 2000).

The mechanisms by which MTs mediate their cardioprotective effects have been examined. MT protection against doxorubicin-induced cytotoxicity was found to be at least partially mediated via the JAK2/STAT3 pathway in murine cardiomyocytes (Rong et al., 2016). MT-induced inhibition of the NF-κB pathway has been linked to prevention of age-associated cardiomyopathy (Cong et al., 2016). A recent study suggests that MT2A protects cardiomyocytes from I/R through p38 inhibition (Zhao et al., 2021 *Preprint*). It has also been shown that MT inhibits doxorubicin-induced mitochondrial cytochrome c release and caspase-3 activation in cardiomyocytes (Wang et al., 2001). Collectively, these studies demonstrate that MTs act to induce the expression of cardioprotective genes and reduce mitochondrial damage due to oxidative stress in cardiac tissue.

### Zinc transporter expression in cardiac dysfunction

In cardiac dysfunction, intracellular Zn^2+^ levels are known to be altered. A role for Zn^2+^ in ischemia was first established in cerebral ischemia in rat brain in 1990 (Tønder et al., 1990) and later demonstrated in isolated rat cardiomyocytes where an ∼30-fold increase in [Zn^2+^]_i_ was observed during ischemia that rapidly decreased upon reoxygenation (Ayaz and Turan, 2006). Hare et al. (2009) observed an accumulation of [Zn^2+^]_i_ in the left ventricle of rat cardiac tissue following I/R.

Alterations in the expression levels of zinc transporters are associated with several cardiovascular events (Table 4). Hara and colleagues suggest that modulation of ZIP13 expression may be important for inflammatory signaling responses in the heart following in vitro treatment with doxorubicin (Hara et al., 2022). In S/ER, ZIP7 and ZnT7 expression is reported to be altered in type 2 diabetes and high glucose conditions, which are both considered risk factors for cardiovascular disease. Protein expression of ZIP7 was significantly decreased while expression of ZnT7 was significantly increased in cardiomyocytes cultured in high glucose conditions and in hearts excised from a diabetic rat model (Tuncay et al., 2019). Tuncay and co-workers also identified significant alterations in ZIP7 and ZnT7 S/ER protein expression in H9C2 cells treated with doxorubicin to simulate heart failure (Tuncay et al., 2017). Furthermore, in cardiac tissue from individuals with heart failure, the expression of ZIP14 and ZnT8 was significantly increased and ZIP8 levels decreased relative to controls (Olgar et al., 2018a). Screening all ZIP and ZnT transporters, Bodiga and colleagues reported alterations in multiple transporters in cardiomyocytes exposed to a hypoxia/reoxygenation protocol, among which were the S/ER-located ZIP7 and ZIP14 transporters (Bodiga et al., 2017).

**Table 4.** Studies examining zinc transporters in cardiovascular disease

Zinc transporter	Experimental model	Protocol	Quantification		Expression change	Reference
			Protein expression	mRNA expression		
ZIP1	CMs isolated from Sprague-Dawley rats (WT, male, 8 wk)	In vivo chronic aldosterone/salt treatment, 4 wk		✓	↑ ∼4.2-fold	Kamalov et al., 2009
	CMs isolated from Wistar Kyoto rats	In vitro H/R	✓		↑ hypoxia 0.5 to ∼1.4 AU ↑ H/R 0.5 to ∼0.7 AU	Bodiga et al., 2017
ZIP2	CMs isolated from Wistar Kyoto rats	In vitro H/R	✓		↑ hypoxia 1 to ∼1.3 AU ↓ H/R 1 to ∼0.8 AU (NS)	Bodiga et al., 2017
	Hearts from C57BL/6 mice (WT, male, 8–10 wk)	In vivo I/R by left anterior descending coronary artery occlusion	✓	✓	↑ protein ∼150% ↑ mRNA ∼fourfold	Du et al., 2019
ZIP3	CMs isolated from Wistar Kyoto rats	In vitro H/R	✓		↑ hypoxia 1 to ∼1.6 AU ↑ H/R 1 to ∼1.6 AU	Bodiga et al., 2017
ZIP6	CMs isolated from Wistar Kyoto rats	In vitro H/R	✓		↑ hypoxia 0.8 to ∼1 AU (NS) ↓ H/R 0.8 to ∼0.7 AU (NS)	Bodiga et al., 2017
ZIP7	CMs isolated from Wistar Kyoto rats	In vitro H/R	✓		↑ hypoxia 1 to ∼2 AU ↓ H/R 1 to ∼0.9 AU (NS)	Bodiga et al., 2017
ZIP7	Hearts from Wistar rats (WT, male, 2 mo)	In vivo transverse aortic constriction	✓		↑ ∼twofold	Olgar et al., 2018b
	H9C2 cell lysates	In vitro DOX treatment	✓		↑ ∼1.5-fold	Tuncay et al., 2019
	CMs isolated from C57BL/6 mice (WT, male, 8–10 wk)	In vitro H/R	✓		↑ ∼0.7 to ∼1.2	Zhang et al., 2021
	Hearts from Wistar rats (WT, male, 250–350 g)	Ex vivo I/R	✓		↑ ∼0.75 to ∼0.9	Zhang et al., 2021
	Hearts from C57BL/6 mice (WT, male, 8–10 wk)	In vivo I/R by left anterior descending coronary artery occlusion	✓	✓	↑ protein ∼0.8 to ∼1 ↑ mRNA from ∼1 to 2	Zhang et al., 2021
ZIP8	H9C2 cell lysates	In vitro DOX treatment	✓		↓ ∼0.4-fold	Olgar et al., 2018a
	Human heart failure tissue	Patients with end-stage heart failure	✓		↓ ∼0.5-fold	Olgar et al., 2018a
	Hearts from Wistar rats (WT, male, 2 mo)	In vivo transverse aortic constriction	✓		↓ ∼0.5-fold	Olgar et al., 2018b
ZIP9	CMs isolated from Wistar Kyoto rats	In vitro H/R	✓		↑ hypoxia 1 to ∼2 AU ≈ H/R	Bodiga et al., 2017
ZIP10	CMs isolated from Wistar Kyoto rats	In vitro H/R	✓		↑ hypoxia 1 to ∼1.5 AU ↑ H/R 1–∼1.2 (NS)	Bodiga et al., 2017
ZIP11	CMs isolated from Wistar Kyoto rats	In vitro H/R	✓		↑ hypoxia 1 to ∼2 AU ≈ H/R	Bodiga et al., 2017
ZIP12	Human pulmonary artery smooth muscle cells	In vitro hypoxia incubation		✓	↑ ∼threefold	Zhao et al., 2015
ZIP13	CMs isolated from Wistar Kyoto rats	In vitro H/R	✓		↑ hypoxia 0.5 to ∼2 AU ≈ H/R	Bodiga et al., 2017
ZIP13	Heart tissue from C57BL/6 mice (WT, male, 8–10 wk)	In vivo left anterior descending coronary artery ligation	✓	✓	↓ protein ∼0.5-fold ↓ mRNA ∼0.6-fold	Wang et al., 2021a
	H9C2 cell lysates	In vitro H/R	✓		↓ ∼0.6-fold	Wang et al., 2021b
	Neonatal CMs isolated from newborn C57BL/6N mice	In vitro DOX treatment		✓	↓ ∼0.75 to ∼0.1	Hara et al., 2022
	Heart tissue from C57BL/6N mice	In vivo intraperitoneal DOX injection		✓	↓ ∼1 to ∼0.6	Hara et al., 2022
ZIP14	CMs isolated from Wistar Kyoto rats	In vitro H/R	✓		↑ hypoxia 0.5 to ∼2 AU ≈ H/R	Bodiga et al., 2017
	H9C2 cell lysates	In vitro DOX treatment	✓		↑ ∼1.5-fold	Olgar et al., 2018a
	Human heart failure tissue	Patients with end-stage heart failure	✓		↑ ∼twofold	Olgar et al., 2018a
	Heart tissue from Wistar rats (WT, male, 2 mo)	In vivo transverse aortic constriction	✓		↑ ∼2.5-fold	Olgar et al., 2018b
ZnT1	Cultures CMs from rats (1–2 d old)	In vitro rapid pacing	✓		↑ 214.4%	Beharier et al., 2007
	Heart homogenates from Sprague-Dawley rats (WT, male, 250–350 g)	In vivo rapid atrial pacing	✓		↑ 148%	Beharier et al., 2007
	Human cardiac tissue	Cardiac tissue obtained from control and atrial fibrillation patients	✓		↑ 0.73–1.88	Etzion et al., 2008
	CMs from Sprague-Dawley rats (WT, male, 8 wk)	In vivo chronic aldosterone/salt treatment, 4 wk		✓	↑ ∼twofold	Kamalov et al., 2009
	CMs isolated from Wistar Kyoto rats	In vitro H/R	✓		↑ hypoxia 1 to ∼2 AU ↑ 1 to ∼1.2 AU (NS)	Bodiga et al., 2017
ZnT2	CMs isolated from Wistar Kyoto rats	In vitro H/R	✓		↑ hypoxia 0.5 to ∼0.6 AU (NS) ↑ H/R 0.4 to ∼1.4 AU	Bodiga et al., 2017
ZnT5	CMs isolated from Wistar Kyoto rats	In vitro H/R	✓		≈ hypoxia ↑ H/R 0.8 to 1.2 AU	Bodiga et al., 2017
ZnT7	Hearts from Wistar rats (WT, male, 2 mo)	In vivo transverse aortic constriction	✓		↓ ∼0.6-fold	Olgar et al., 2018b
	H9C2 cell lysates	In vitro DOX treatment	✓		↓ ∼0.5-fold	Tuncay et al., 2019
ZnT8	H9C2 cell lysates	In vitro DOX treatment	✓		↑ ∼1.6-fold	Olgar et al., 2018a
	Human heart failure tissue	Patients with end-stage heart failure	✓		↑ ∼twofold	Olgar et al., 2018a
	Hearts from Wistar rats (WT, male, 2 mo)	In vivo transverse aortic constriction	✓		↑ ∼1.5-fold	Olgar et al., 2018b
ZnT9	CMs isolated from Wistar Kyoto rats	In vitro H/R	✓		↑ hypoxia 0.8 to ∼1 AU (NS) ↑ H/R 0.8 to ∼1.1 AU (NS)	Bodiga et al., 2017

Changes observed in ZIPs and ZnTs in conditions of cardiovascular disease including experimental model, expression change, and study. All expression changes are significant except where NS (not significant) is specified. CMs, cardiomyocytes; DOX, doxorubicin; H/R, hypoxia/reoxygenation. ↑ denotes increased expression; ↓ illustrates a decrease in expression; ≈ shows no change.

### Zn^2+^ dyshomeostasis in EC coupling

The importance of tightly controlled cellular Zn^2+^ homeostasis for the prevention of cardiac dysfunction is beginning to emerge (Alvarez-Collazo et al., 2012; Turan and Tuncay, 2017). In animal models, dysregulated levels of intracellular Zn^2+^ are associated with severe cardiac degeneration in Duchenne muscular dystrophy (Crawford and Bhattacharya, 1987). Male mice deficient in ZnT5 have significantly higher frequency of bradyarrhythmias and mortality rate compared with control animals (Inoue et al., 2002). Also, Zn^2+^ significantly contributes to oxidant-induced alterations of EC coupling (Turan et al., 1997). Defective Zn^2+^ handling contributes to the cellular pathology of certain cardiomyopathies, including altered contractility and heart failure (Kleinfeld and Stein, 1968; Kalfakakou et al., 1993; Little et al., 2010). The underlying mechanism of how Zn^2+^ contributes to these pathologies is still not fully understood. Cytosolic Zn^2+^ has recently been shown to act as a high-affinity activator of RyR2, able to activate channels even when [Ca^2+^]_i_ is subactivating (Woodier et al., 2015; Reilly-O’Donnell et al., 2017) providing an important mechanistic explanation for how Zn^2+^ dyshomeostasis can result in altered Ca^2+^ dynamics and cardiac dysfunction. An emerging and important research area is therefore to understand how altered Zn^2+^ levels evoke deleterious effects on cardiac functioning.

### Zn^2+^ dyshomeostasis in cardiac morphogenesis

Zinc transporters are of key importance in embryonic development and cardiac morphogenesis. Knockout of ZnT1 or ZIP7 is embryonically lethal (Andrews et al., 2004; Woodruff et al., 2018). Knockout of ZIP8 is also embryonically lethal in mice with hypertrabeculation and noncompaction of the ventricles observed, while knockdown of ZIP10 in zebrafish results in heart deformities (Taylor et al., 2016; Lin et al., 2018). Additionally, recent research shows that primary neonatal cardiomyocytes from ZIP13 knockout mice display arrhythmic beating (Hara et al., 2022).

The findings of Inoue and colleagues are also noteworthy, where ZnT5 knockout resulted in male-specific sudden death from bradyarrhythmia (Inoue et al., 2002). Loss-of-function mutation of ZnT5 is reported to result in lethal cardiomyopathy and premature death in a case study by Lieberwirth et al. (2021). This illustrates that zinc transporters as well as calcium channels are necessary in cardiac development and function.

### Zn^2+^ dyshomeostasis as a new pharmacological target in cardiovascular disease

Sacubitril/valsartan (formally known as LCZ696) is an active substance in the drug Entersto, which is used to treat chronic heart failure (Khalil et al., 2018). Sacubitril/valsartan is an angiotensin II type 1 receptor blocker that inhibits neprilysin and is currently being trialed for treatment of patients with chronic systolic heart failure (ClinicalTrials.gov identifier: NCT01035255; McMurray et al., 2013). These trials are of interest as neprilysin is a zinc-dependent plasma membrane type II integral protein metallopeptidase which contains a Zn^2+^-binding site on its extracellular C-terminal domain (Fulcher and Kenny, 1983; Nalivaeva et al., 2020), linking Zn^2+^ dependent processes with cardiovascular function.

There have also been trials examining the usefulness of Zn^2+^ chelation. The TACT trial (NCT00044213) investigated the effect of chelation therapy using EDTA on the occurrence of subsequent cardiovascular events in participants with previous myocardial infarction (Lamas et al., 2013). EDTA is a chelator of not only Zn^2+^ but also of Ca^2+^, Mg^2+^, Fe^2+^/Fe^3+^, Cd^2+^, and Cu^2+^ (Lamas et al., 2013). Reactive binding of EDTA to metals is as follows: Cr^2+^ >Fe^3+^ >Cu^2+^ >Pb^2+^ >Zn^2+^ >Cd^2+^ >Co^2+^ >Fe^2+^ >Mn^2+^ >Ca^2+^ >Mg^2+^, therefore, EDTA will preferentially bind Zn^2+^ (estimated *K*_d_ 10^-16^ M) over other divalent metals in plasma including Ca^2+^ (*K*_d_ ∼10^−11^ M) due to the high affinity EDTA has for Zn^2+^ (Waters et al., 2001; commentary by Nyborg and Peersen, 2004). The trial concluded that treatment with EDTA modestly reduced the risk of adverse cardiovascular outcomes. However, the evidence was not sufficient to justify the implementation of chelation therapy as a routine postmyocardial infarction treatment (Lamas et al., 2013). The research has been continued in the TACT2 trial, which is focusing on chelation therapy in patients with diabetes who have had a previous myocardial infarction (NCT02733185; U.S. National Library of Medicine, 2022). This trial is due for completion in December 2023 (U.S. National Library of Medicine, 2022). The targeting of Zn^2+^ to improve patient outcome in myocardial infarction and heart failure has not yet resulted in development of new cardiovascular disease treatments. In addition, Zn^2+^ levels cannot be used as a biomarker for cardiovascular disease as several factors including dietary intake and blood glucose levels can alter plasma Zn^2+^ concentration and zinc handling (Fernández-Cao et al., 2019). However, it is possible that chelation of Zn^2+^ in the short term, for example, during myocardial infarction, would help to attenuate the damage observed postmyocardial infarction.

## Concluding remarks

The role of ZIPs, ZnTs, and Zn^2+^-binding proteins in the heart provides novel insights into the regulation of cellular Zn^2+^ and its role as a signaling molecule in cardiac tissue. The ability of Zn^2+^ to act as a regulator and/or activator of cellular Ca^2+^ channels suggests a new and important role for Zn^2+^ in cardiac function under both physiological and pathological conditions, raising the suggestion that correction of Zn^2+^ dyshomeostasis may be a novel therapeutic strategy to combat cardiovascular diseases.

In comparison to Ca^2+^, there has been relatively little work investigating the biological function of Zn^2+^ in the heart. Consideration of accurate [Zn^2+^]_i_ measurements should be emphasized as failure to acknowledge dynamic Zn^2+^ changes could lead to significant overestimation of [Ca^2+^]_i_. Indeed, many of the tools routinely used to measure Ca^2+^ also bind Zn^2+^, challenging us to consider how many processes driven by Ca^2+^ may also be in part, attributable to Zn^2+^ (Stork and Li, 2006; Figueroa et al., 2014; Fujikawa et al., 2015). Thanks to the development of appropriate tools enabling us to accurately monitor Zn^2+^ fluxes and the ability of these methods to distinguish Zn^2+^ from Ca^2+^ in biological systems, the field of zinc biology is currently advancing rapidly (for a comprehensive overview of different Zn^2+^ sensors, see Huang and Lippard, 2012; Carpenter et al., 2016; Pratt et al., 2021). Much has been learned relating to the intrinsic relationships that exist between Zn^2+^ and Ca^2+^ homeostatic mechanisms and their roles in heart disease. However, more work is needed to fully understand the role of Zn^2+^ in the heart. This includes better understanding of cellular Zn^2+^ dynamics, how Zn^2+^ is regulated, and the biological targets of labile Zn^2+^. This will require a greater appreciation of the spatio-temporal patterning of intracellular Zn^2+^ fluxes in the heart and how these relate to cardiac functioning in health and disease.
